# Curvilinear relationship between burnout and work engagement among staff in community services for the elderly: A correlation study

**DOI:** 10.3389/fpubh.2022.939649

**Published:** 2022-07-22

**Authors:** Guangmei Yang, Huiyan Wei, Leping Wan, Haiying Dong, Xiaoxiao Liang, Yan He

**Affiliations:** Department of Social Medicine and Health Care Management, School of Public Health, Zhengzhou University, Henan, China

**Keywords:** curvilinear relationship, burnout, work engagement, community services for the elderly, staff

## Abstract

**Objective:**

To investigate whether there is a curvilinear relationship between burnout and work engagement among staff in Chinese community services for the elderly.

**Methods:**

A stratified whole-group random sampling method was used to survey 244 staff members from eight communities in two cities. Data were collected using the Maslach Burnout Inventory scale (MBI) and the Utrecht Work Engagement Scale- 9 (UWES- 9). The curve estimation method explored the functional model of burnout and work engagement scales.

**Results:**

Two hundred forty-four staff members completed the survey. Burnout, depersonalization (DP), and personal accomplishment (PA) were found to be related to work engagement in a cubic function (*R*^2^ = 0.166, *P* < 0.05), (*R*^2^ = 0.061, *P* < 0.05), and (*R*^2^ = 0.2230, *P* < 0.05), respectively. There was no statistically significant relationship between emotional exhaustion (EE) and work engagement (*P* > 0.05). “Personal Accomplishment” is related to work engagement in a U-shaped curve.

**Conclusions:**

There was a cubic function relationship between burnout and work engagement, where “personal accomplishment” was related to work engagement in a U-shaped curve. Therefore, the government and related service organizations should understand the impact of different levels of burnout on work engagement and take targeted measures to alleviate burnout and improve work engagement by targeting emotions and stroke.

## Introduction

In China, as the number of elderly people increases and the demand for elderly services increases, society demands more staff for community services for the elderly. As a result, the Chinese government and related service organizations pay more attention to staff burnout in community services for the elderly and place higher demands on their work engagement. The staff needs to reduce burnout and increase engagement to ensure the elderly satisfaction and build harmonious relationships with the elderly. Burnout is a negative emotional response to long-term work stress that can directly or indirectly affect workers' mental and physical health (1). Burnout includes emotional exhaustion (EE), depersonalization (DP), and personal accomplishment (PA), a concept first introduced by Herbert Freudenberger (2, 3), who used it to describe negative emotions that arise under prolonged work (4). Maslach et al. (5) further classified burnout into three categories or domains. EE represents excessive depletion of one's emotions resulting in a low mood. DP represents a negative and indifferent attitude toward people and things at work. PA represents a lack of self-confidence in the self-evaluation dimension and a lack of value at work. The Burnout scale developed by Maslach et al. (5) has been validated in China on its excellent reliability and validity (6, 7). Burnout has been used more often in the teaching field (8–10), where studies have shown that the main reason for leaving the teaching profession is burnout (11). Burnout is not only applied to education but is now considered an epidemic in the health field. A large body of literature examines the phenomenon and factors of burnout among health professionals in different specialties, including mainly surgeons (12), family physicians (13), general practitioners (14), dentists (15), and others. However, fewer researches have been conducted on staff who serve the elderly. As aging increases, burnout among staff in the community is gaining attention because it hurts staff health (16) and takes a toll on the quality of services for the elderly (17, 18). At the same time, burnout can affect the accuracy of decisions made by staff (19). Work engagement is a positive, joyful mindset at work. Schaufeli et al. (20) explained work engagement as approaching work with positive, fulfilling emotions, consisting primarily of vigor, dedication, and absorption. Among other things, “vigor” means the ability to put more energy into the work and not to give up lightly. “Dedication” refers to the commitment to the work and the use as a challenge. “Absorption” means to concentrate on something to achieve a satisfactory state. Work engagement is a hot topic in multiple fields (21). In addition to positively affecting staff competence (22), increased staff engagement in a community can also increase the satisfaction of the elderly (17, 23). Elderly care is a profession that involves a lot of emotion, and staff members can't help but show empathy in caring for the elderly (24). And the staff are prone to burnout during extended periods with the elderly (18, 25). Continued burnout inevitably affects staff work engagement. So far, most studies have concluded a negative correlation between the two (26, 27). But work engagement is not only affected by burnout but also by psychological conditions (28), workplace bullying (29), work value perception (30), work-family balance (31), active motivation, passive motivation and demotivation (32). One such study of teachers by Pishghadam et al. (33, 34) found that when teachers' initiative is reduced, they become disillusioned with continuing their education and can suffer from learning burnout. Although teachers' motivation is affected by burnout, due to other factors, such as teacher experience, they will learn how to cope with the negatives and will in turn influence their commitment to their work (35). This also applies to staff working in community services for the elderly.

In addition, the dissatisfaction of the elderly with staff caused by burnout or the lack of diligence noted by supervisors can stimulate staff to work hard (36). This is seen as motivation, which is defined as the factors that drive staff to choose or continue in their careers (37), i.e., when staff are less engaged in their work, subject to institutional interventions, systems, and regulations that have a positive impact on work engagement These extrinsic incentives also include workload (38), financial resources (39), government policies (40), etc. Therefore, burnout and work engagement may have not only a linear relationship but also a curvilinear relationship. This study was conducted to explore a curvilinear relationship between the two.

## Materials and methods

### Settings and participants

**Figure 1:**
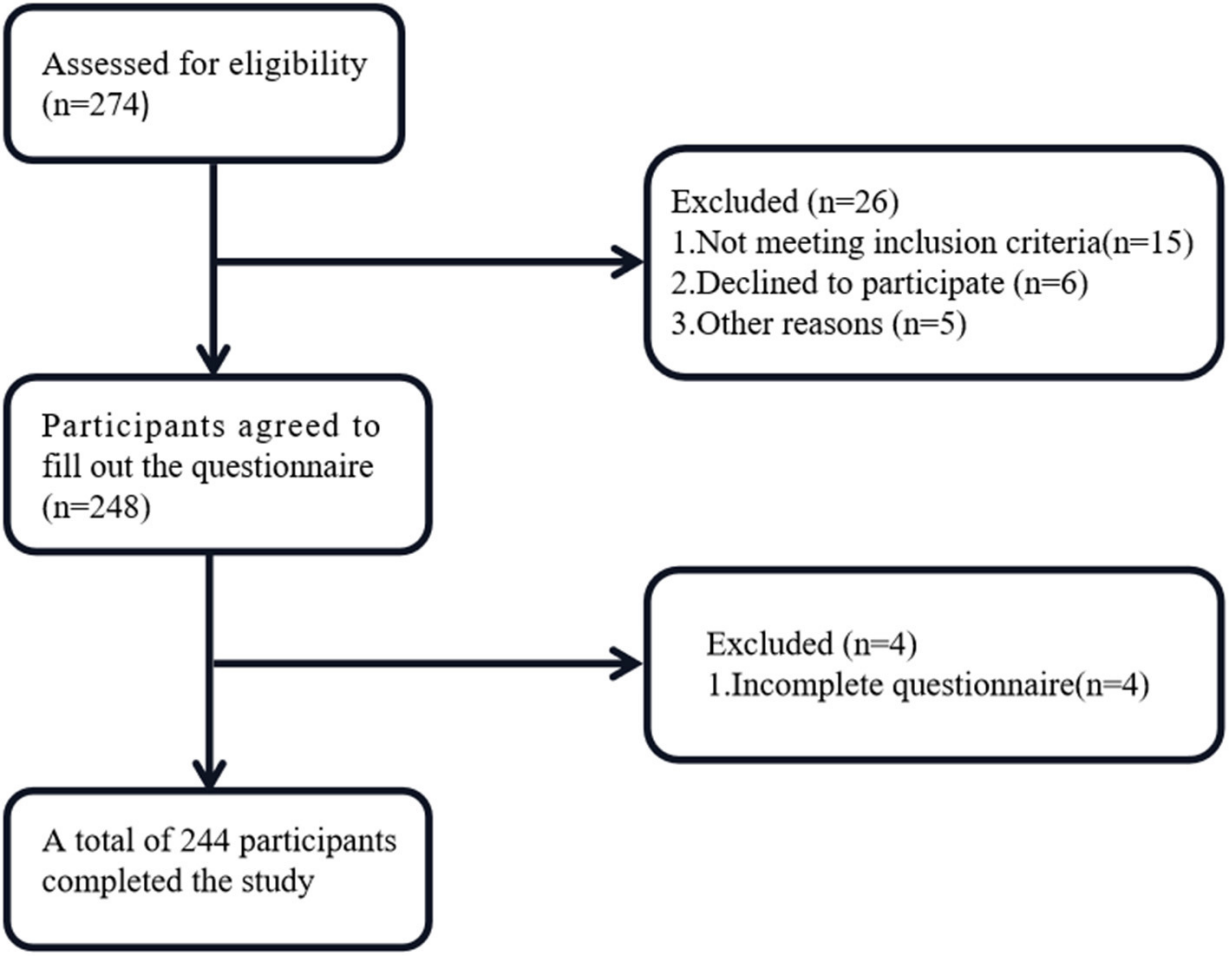
Flowchart on participant recruitment.

This study conducted a questionnaire survey with community staff in two cities, Guangzhou and Suzhou, from September to October 2021. This study used a multi-stage sampling method to ensure that the sample was representative. In the first stage, two provinces were randomly selected from 12 provinces in the eastern region, namely Jiangsu Province and Guangdong Province. Secondly, one municipality was randomly selected from within each province. Suzhou in Jiangsu Province and Guangzhou in Guangdong Province. In the second stage, a county/district was randomly selected within the jurisdiction of each city. Suzhou was chosen as Kunshan County, and Guangzhou was chosen as Yuexiu District. Next, two streets were randomly selected from each city/district. Finally, four communities were randomly selected from each street. In the third stage, staff were randomly selected from each community. Two hundred and forty four questionnaires were collected from 274 staff in eight elderly communities, excluding those with logical errors and missing data, with an effective response rate of 89.05%. Among the participants, 169 (69.3%) were surveyed in Suzhou, and 75 (30.7%) were surveyed in Guangzhou. The participant recruitment process is shown in Figure 1.

### Measurement

#### The general condition questionnaire

Using self-designed basic information questionnaire (Chinese version), including (1) Basic personal information: gender, age, education, salary, title, etc. (2) Work situation: promotion and salary increase time, continuing education, job satisfaction, career prospects, etc.

#### The maslach burnout inventory scale (MBI)

The MBI, developed by Maslach et al. (5) and revised by Chinese translation, includes 15 - items in 3 dimensions. A 7-point Likert scale ranged from 0 (never) to 6 (every day). The total burnout score is the sum of the mean scores of each dimension, i.e., total burnout score = emotional exhaustion ^*^ 0.4 + depersonalization ^*^ 0.3 + personal accomplishment ^*^ 0.3. The Cronbach's alpha coefficient of this scale in this study was 0.697, which effectively assessed the burnout level of staff in the community.

#### The utrecht work engagement scale- 9 (UWES- 9)

The UWES- 9, developed by Schaufeli et al. (20) and with reduced entries, contains 9- items in 3 dimensions. The scale uses a 7-point Likert scale ranging from 0 (never) to 6 (every day). The higher the score, the higher the staff's work engagement. The scale has a Cronbach's alpha coefficient of 0.876 with good reliability.

### Data analysis

SPSS 21.0 was used for data analysis, normally distributed measures were expressed as ($\bar{x}$ ± s), count data were expressed as a rate (%), and the two test was used to compare groups. Pearson correlation was used to analyze the correlation between burnout and work engagement dimensions. The test level was α = 0.05. A mathematical model to predict the relationship between burnout and work engagement was developed through regression analysis. A curvilinear regression model was developed with “work engagement” as the dependent variable. Burnout and its three components (“emotional exhaustion,” “depersonalization,” and “personal accomplishment”) as the independent variables. The curve that best fits the data is selected by calculating the model that produces the highest coefficient of determination. Considering the significant differences between the data from the two regions, we extracted the data from the stratified sample, figured the data for both cities and performed a comparative analysis.

## Results

### Descriptive statistics

The percentage of staff in each variable is shown in Table 1. Among them, different regions, education, title, years of work, job satisfaction and career prospects influenced burnout and work engagement (*P* < 0.05). Different age, salary and continuing education influenced burnout (*P* < 0.05). The timing of different promotions and salary increase was a factor influencing work engagement (*P* < 0.05).

**Table 1:** Basic information about the participants.

**Descriptive characteristics**	***N***	**(%)**	**Burnout**		**Work engagement**	
			**Score**	***P***	**Score**	***P***
**Region**				<0.001		<0.001
Suzhou	169	69.3	1.437 ± 0.529		11.458 ± 3.267	
Guangzhou	75	30.7	8.600 ± 3.520		9.387 ± 3.434	
**Gender**				0.542		0.623
Male	58	23.8	3.360 ± 3.418		10.626 ± 3.906	
Female	186	76.2	3.018 ± 3.525		10.882 ± 3.301	
**Age (years)**				0.017		0.087
<30	80	32.8	2.971 ± 3.416		11.488 ± 3.327	
30~45	50	20.5	4.409 ± 4.902		11.047 ± 3.479	
45~60	93	38.1	2.929 ± 2.968		10.176 ± 3.535	
>60	21	8.6	1.595 ± 0.647		10.603 ± 3.108	
**Education**				0.002		<0.001
Undergraduate or above	28	11.5	2.766 ± 2.860		11.143 ± 3.598	
High School/Junior College	139	57.0	2.478 ± 2.863		11.743 ± 3.185	
Secondary school/junior high school and below	77	31.5	4.276 ± 4.328		9.039 ± 3.188	
**Salary (yuan)**				0.001		0.835
<3,500	95	38.9	2.073 ± 2.070		10.972 ± 3.577	
3,500–5,500	103	42.2	3.604 ± 4.085		10.774 ± 3.696	
>5,500	46	18.9	4.135 ± 3.970		10.616 ± 2.528	
**Title**				<0.001		<0.001
Intermediate and above	19	7.8	2.080 ± 1.956		12.983 ± 3.762	
Elementary	65	26.6	2.881 ± 3.501		11.862 ± 3.003	
Other	47	19.3	6.539 ± 4.345		9.028 ± 3.335	
None	113	46.3	2.067 ± 2.357		10.605 ± 3.312	
**Years of work/year**				<0.001		0.047
<2	120	49.2	2.849 ± 3.407		11.189 ± 3.587	
2–4	62	25.4	2.073 ± 2.223		11.124 ± 3.210	
4–6	35	14.3	3.681 ± 3.773		10.124 ± 2.758	
>6	27	11.1	6.618 ± 4.466		9.395 ± 3.801	
**Promotion and salary increase time/year**				0.053		0.049
≤ 1	94	38.5	3.939 ± 4.016		11.192 ± 3.272	
1-~5	22	9.0	2.324 ± 2.324		10.591 ± 3.091	
Subject to merit	91	37.3	2.538 ± 3.325		11.066 ± 3.620	
Other	37	15.2	3.342 ± 3.171		9.414 ± 3.414	
**Continuing education**				<0.001		0.104
Not accepted	79	32.4	5.033 ± 4.316		10.135 ± 3.964	
Provincial	34	13.9	1.970 ± 1.468		11.275 ± 2.936	
Municipal	44	18.0	2.292 ± 2.807		10.606 ± 3.025	
County and district level	87	35.7	1.904 ± 2.301		11.376 ± 3.256	
**Job satisfaction**				<0.001		<0.001
Very satisfied	25	10.2	1.749 ± 3.345		12.893 ± 3.149	
Relatively satisfied	132	54.1	2.342 ± 2.354		11.404 ± 3.281	
Fair	87	35.7	4.772 ± 4.434		9.341 ± 3.229	
**Career prospects**				0.001		<0.001
Very good	32	13.1	1.657 ± 2.884		14.708 ± 2.666	
Better	107	43.9	2.602 ± 2.807		10.798 ± 3.216	
Fair	105	43.0	4.009 ± 3.984		9.660 ± 1.081	

### Correlation analyses

Most study participants had a negative correlation between burnout and work engagement dimensions (*P* < 0.05). See Table 2.

**Table 2:** Correlation analysis between burnout and work engagement.

**Classification**	**Work engagement**	**Vigor**	**Dedication**	**Absorption**
Burnout	−0.327**	−0.334**	−0.305**	−0.243**
EE	−0.090	−0.043	−0.161*	−0.051
DP	−0.236**	−0.217**	−0.306**	−0.127*
PA	−0.378**	−0.312**	−0.346**	−0.362**

^**^P < 0.01; ^*^P < 0.05.

### Curvilinear relationships

**Figure 2:**
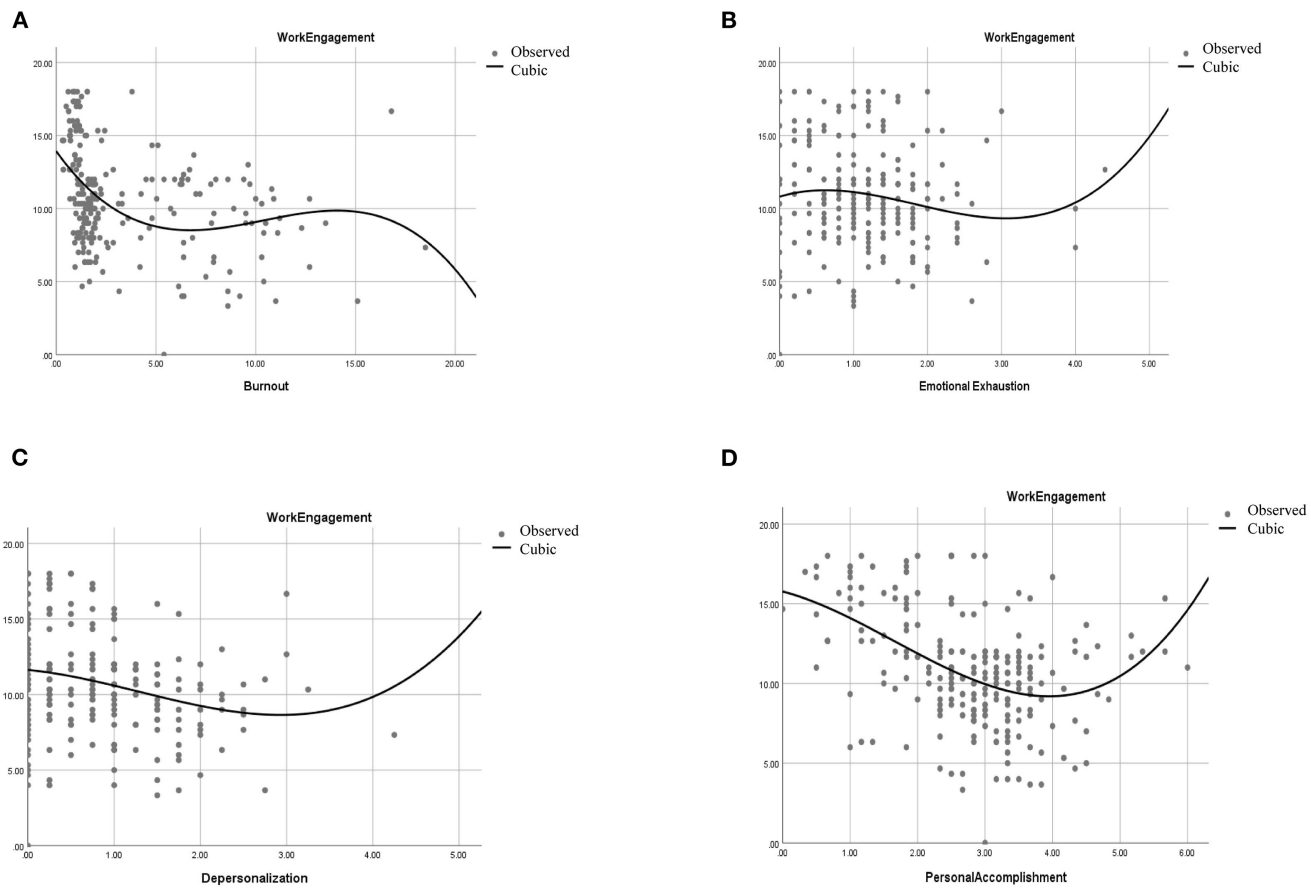
Curvilinear relationships. **(A)** Curve relationships between work engagement and burnout. **(B)** Curve relationship between work engagement and emotional exhaustion. **(C)** Curve relationship between work engagement and depersonalization. **(D)** Curve relationship between work engagement and personal accomplishment.

The curve estimation results show that constructing a cubic function model with work engagement as the dependent variable and burnout as the independent variable is the best-fit model (*R*^2^ = 0.166, *P* < 0.05). The models constructed in different regions are not quite the same. The cubic function model in the Suzhou region is the best, and the inverse function model in the Guangzhou region is the best, as shown in Model formula 1, See Table 3. The curve function model is shown in Figure 2 (Based on the all data, Figure 2A).

**Table 3:** Curve relationship.

**Model formula**	**Suzhou**	**Guangzhou**	**Total**
Model formula 1	Y1 = 18.360-6.914X_1_+0.999X${}_{1}^{2}+$0.165X_1_	Y1 = 8.094+8.003/X_1_	Y1 = 13.933-0.918X_1_+0.211X${}_{1}^{2}$-0.007X${}_{1}^{3}$
*R^2^*	0.222	0.222	0.166
*P*	<0.001	0.041	<0.001
Model formula 2	Y2 = 12.247-0.318X_2_-0.510X${}_{2}^{2}+$0.132X${}_{2}^{3}$	Y2 = 9.019+0.402X_2_+0.054X${}_{2}^{2}$-0.053X${}_{2}^{3}$	Y2 = 10.817+1.509X_2_-1.463X${}_{2}^{2}+$0.265X${}_{2}^{3}$
*R^2^*	0.025	0.006	0.017
*P*	0.245	0.940	0.258
Model formula 3	Y3 = 11.979+0.634X_3_-2.102X${}_{3}^{2}+$0.585X${}_{3}^{3}$	Y3 = 9.943-0.034X_3_+0.507X${}_{3}^{2}$-0.108X${}_{3}^{3}$	Y3 = 11.628-0.541X_3_-0.671X${}_{3}^{2}+$0.174X${}_{3}^{3}$
*R^2^*	0.052	0.022	0.061
*P*	0.032	0.534	0.002
Model formula 4	Y4 = 14.856+0.972X_4_-1.463X${}_{4}^{2}+$0.219X${}_{4}^{3}$	Y4 = 17.547-7.439X_4_+1.868X${}_{4}^{2}$-0.128X${}_{4}^{3}$	Y4 = 15.763-1.097X_4_-0.709X${}_{4}^{2}+$0.143X${}_{4}^{3}$
*R^2^*	0.287	0.174	0.230
*P*	<0.001	0.003	<0.001

*Model formula 1 Curve relationship between work engagement and burnout*.

*Model formula 2 Curves relationship between work engagement and emotional exhaustion*.

*Model formula 3 Curve relationship between work engagement and depersonalization*.

*Model formula 4 Curve relationship between work engagement and personal accomplishment*.

The curve estimation results showed that the fitted model constructed with work engagement as the dependent variable and “emotional exhaustion” as the independent variable was not statistically significant (*P* > 0.05), as shown in Model formula 2, See Table 3. The curve function model is shown in Figure 2 (Based on the all data, Figure 2B).

The best-fit model was constructed as a cubic function model with work input as the dependent variable and “depersonalization” as the independent variable (*R*^2^ = 0.061, *P* < 0.05), as shown in Model formula 3, See Table 3. The curvilinear function model is shown in Figure 2 (Based on the all data, Figure 2C).

Finally, with work engagement as the dependent variable and “personal accomplishment” as the independent variable, the best-fit model was constructed as a cubic function model (*R*^2^ = 0.230, *P* < 0.05), as shown in Model formula 4, See Table 3. The highest *R*^2^ (0.287) was found in Suzhou, and the graph of the curve function is shown in Figure 2 (Based on the all data, Figure 2D).

## Discussion

Burnout is a kind of negativity that occurs among the staff in the community who serve the elderly. Work engagement determines the degree of attention to the elderly in their work and the satisfaction of the elderly in receiving services. Unlike previous studies, this study explored the relationship between burnout and the curve of work engagement for the first time. A stratified whole-group random sample was used to sample staff from institutions in different economic regions, increasing the diversity and representativeness of the model.

The results of the correlation analysis study in this study showed that there was a significant negative correlation between burnout and work engagement. The results of the simultaneous curve estimation show that the mathematical model of the cubic function between these two variables fits best. As found in Figure 2A, within the range values of burnout, the value of about 5 and about 15 points of burnout divided the curve into three segments. Work engagement increased with burnout in both intervals between 0 and 5 and after 15 points, similar to the usual findings (41–43). On a scale between 5 and 15, work engagement increases with burnout, which means that an increase in burnout instead increases work engagement. This may be because burnout reaches a certain point where it attracts the attention of other relevant leaders, who use external monitoring to push staff to take their work seriously. However, it is assumed that staff will grow weary of this supervision, and their commitment to their work will again diminish. Such supervisory hygiene factors do not guarantee staff commitment in the long term, and motivational factors are needed to motivate staff intrinsically.

The curve between work engagement and “depersonalization” and “personal accomplishment” has a U-shaped relationship. The study results found that depersonalization scores around three were negatively correlated with work engagement, and after three were positively correlated with work engagement. However, the sample size is smaller after three points. A small number of incidental responses may significantly alter the shape of the curve in the high-scoring area. Therefore, the curve between work engagement and “depersonalization” is not very strong, but a linear correlation can be established. This is consistent with the findings of Taycan et al. (44) that “depersonalization” affects work engagement. After a score of 4 for personal accomplishment, work engagement increases as the score for personal accomplishment increases. This indicates that a certain degree of “personal accomplishment” also motivates staff to work engagement. In their studies, Wu et al. (45) showed that low achievement affects work demands. Van den Berg JW (46) et al. showed that happiness affects work engagement. Van den Berg JW et al. studied happiness as similar to emotional changes. Emotions are defined as relatively cognitive sensory induced emotions (47). Individuals' emotions are influenced by various internal and external environmental factors, and this shift can be forward and backward and will be completed over time (48). Pishghadam et al. (33, 34, 49) argue that individuals can be involved (hearing and seeing something) and involved (direct experience of something) according to emotion and that the level of emotion seems to affect motivation. All of these studies may suggest that emotions affect work engagement, and this could equally be argued for a sense of accomplishment to affect work engagement. Still, there is no relevant research that indicates that reduced achievement has some positive effect on work engagement, and more research is needed to verify this.

In addition to focusing on staff emotions to increase motivation, this can also be achieved by meeting their own needs. Stroke means paying attention to your needs, strokes can be positive/negative, verbal/non-verbal, and conditional/unconditional (50). Some studies have shown that stroke promotes good student performance (51) and that there is a significant positive correlation between stroke and achieving success (8, 50, 52). The individual's psychological need for stroke is a consequence of what they have done, not an unconditional acceptance of it (32, 50, 52). Thus, acceptance of stroke potentially increases positive motivation and ultimately increases the perception of staff success, which in turn increases staff motivation and to some extent also alleviates burnout.

This study was conducted on community staff in two regions with significant economic differences, and the findings are somewhat generalizable. Secondly, this study is innovative in that it is a survey of the community staff, for which there have been fewer studies in previous research. However, this survey is a questionnaire filled out by the staff according to their situation, easily influenced by personal subjectivity. In addition, the curvilinear relationship between job burnout and work engagement has not been explored in other relevant literature, and the reader is unable to analyze the findings with other relevant literature critically, and more research is still needed to follow to verify the accuracy of this paper.

## Conclusions

The curvilinear relationship between burnout and work engagement found in this study suggests that the government and related service organizations need to use a variety of approaches to help staff alleviate burnout and increase their work engagement. The study results show a cubic function between burnout and work engagement and that “personal accomplishment” is related to work engagement in a U-shaped curve. Therefore, the government and related service organizations should understand the impact of different levels of burnout on work engagement and take targeted measures to alleviate burnout and improve work engagement by targeting emotions and stroke.

## Data availability statement

The original contributions presented in the study are included in the article/supplementary materials, further inquiries can be directed to the corresponding author.

## Ethics statement

The studies involving human participants were reviewed and approved by Ethics Committee of Zhengzhou University (Approval No. ZZUIRB2022-07). The patients/participants provided their written informed consent to participate in this study.

## Author contributions

YH received the grant, participated in the interpretation of the results and critical revision of important intellectual content of the manuscript, and approved the final version of the manuscript. GY and YH designed the study. GY wrote the main manuscript text. LW, HD, and XL collected the data. GY and HW analyzed the data. All authors reviewed the manuscript.

## Funding

This study was supported by National Key Research and Development Program Projects of China (2020YFC2006100) and 2021 Henan Province Science and Technology Research Project (212102310814).

## Conflict of interest

The authors declare that the research was conducted in the absence of any commercial or financial relationships that could be construed as a potential conflict of interest.

## Publisher's note

All claims expressed in this article are solely those of the authors and do not necessarily represent those of their affiliated organizations, or those of the publisher, the editors and the reviewers. Any product that may be evaluated in this article, or claim that may be made by its manufacturer, is not guaranteed or endorsed by the publisher.
